# A multistage mixed methods study protocol to evaluate the implementation and impact of a reconfiguration of acute medicine in Ireland’s hospitals

**DOI:** 10.1186/s12913-019-4629-5

**Published:** 2019-10-29

**Authors:** E. Hurley, S. McHugh, J. Browne, L. Vaughan, C. Normand

**Affiliations:** 1 Centre for Health Policy and Management, Trinity College Dublin, Dublin, Ireland; 2 School of Public Health, University College Cork, Cork, Ireland; 3 Nuffield Trust, London, UK

**Keywords:** Acute medicine, Acute medical unit, System reconfiguration, Programme implementation, Mixed methods

## Abstract

**Background:**

To address deficits in the delivery of acute services in Ireland, the National Acute Medicine Programme (NAMP) was established in 2010 to optimise the management of acutely ill medical patients in the hospital setting, and to ensure their supported discharge to primary and community-based care. NAMP aims to reduce inappropriate hospital admissions, reduce length of hospital stay and ensure patients receive timely treatment in the most appropriate setting. It does so primarily via the development of Acute Medical Assessment Units (AMAUs) for the rapid assessment and management of medical patients presenting to hospitals, as well as streamlining the care of those admitted for further care. This study will examine the impact of this programme on patient care and identify the factors influencing its implementation and operation.

**Methods:**

We will use a multistage mixed methods evaluation with an explanatory sequential design. Firstly, we will develop a logic model to describe the programme’s outcomes, its components and the mechanisms of change by which it expects to achieve these outcomes. Then we will assess implementation by measuring utilisation of the Units and comparing the organisational functions implemented to that recommended by the NAMP model of care. Using comparative case study research, we will identify the factors which have influenced the programme’s implementation and its operation using the Consolidated Framework for Implementation Research to guide data collection and analysis. This will be followed by an estimation of the impact of the programme on reducing overnight emergency admissions for potentially avoidable medical conditions, and reducing length of hospital stay of acute medical patients. Lastly, data from each stage will be integrated to examine how the programme’s outcomes can be explained by the level of implementation.

**Discussion:**

This formative evaluation will enable us to examine whether the NAMP is improving patient care and importantly draw conclusions on how it is doing so. It will identify the factors that contribute to how well the programme is being implemented in the real-world. Lessons learnt will be instrumental in sustaining this programme as well as planning, implementing, and assessing other transformative programmes, especially in the acute care setting.

## Background

Ireland, as with other jurisdictions [1, 2], has seen a significant reduction in its acute beds with a 13% reduction in in-patient beds between 2007 and 2012 [3], and has a large unmet demand for long term care beds [3, 4]. This situation, along with continued growth in demand for emergency services, is resulting in patients waiting longer in overcrowded Emergency Departments (EDs) [5–9], and often receiving suboptimal care on trolleys and wards which are not fit for purpose [10, 11]. In view of this increased demand and reduced capacity, hospitals are finding innovative ways to make better use of existing bed stock by implementing interventions to reduce avoidable admissions, reduce variations in length of stay and improve the safe discharge of patients [1]. The development of the discipline of Acute Medicine and the introduction of Acute Medical Units (AMUs) is seen as one such approach to manage the rates of increase [12]. An AMU is defined as ‘... *a dedicated facility within a hospital that acts as the focus for acute medical care for patients who have presented as medical emergencies to hospital or who have developed an acute medical illness while in hospital’* [13]. These Units are also known in other jurisdictions as Acute Medical Assessment Units (AMAUs), Medical Assessment Units (MAUs), Acute Assessment Units (AAUs), Medical Assessment and Planning Units (MAPUs), and Admission and Planning Units (APUs). While there is wide variation in how these Units are designed and operated, it is recommended that they are co-located on the same floor with other acute and emergency services, and are staffed by acute medicine physicians or specialist consultants with an interest in acute medicine. It is expected that the presence of a senior decision maker expedites the clinical decision making process and improves patient care by facilitating timely review of each patient as they arrive in the Unit [14]. This model of acute care delivery has been adopted in the UK, Australia and New Zealand [15, 16], and more recently the Netherlands [17]. The majority of medical patients presenting to hospitals as emergencies in the UK are now assessed and treated in AMUs, either directly, or after triage in an Emergency Department [18], and these Units are considered essential for improving the quality of care for patients presenting to hospitals with complex medical conditions [19, 20].

### The Irish National Acute Medicine Programme

The National Acute Medicine Programme (NAMP) was introduced in Ireland in 2010 to provide a framework for the delivery of acute medical services and to address deficits in the care of acutely ill medical patients presenting as emergencies to Irish hospitals [21]. Central to the programme was the development of AMUs in all major hospitals, and similar functioning, but smaller AMAUs in smaller hospitals. [A note on terminology used in this study: a fully functioning AMU consists of an AMAU with an associated short stay ward (SSW) for patients whose length of hospital stay is not expected to be greater than 48 h. For consistency, we will refer to Units in the Irish setting as AMAUs, and identify those with an SSW]. As with the UK model, the purpose of these Units is to facilitate the streaming of medical patients either directly from GPs or from ED at triage, into a designated assessment area where they will be rapidly assessed and diagnosed by a senior decision maker (a consultant physician or a registrar/specialist registrar) within a 1 h target and the decision made within a 6 h target to discharge home, admit to an adjacent short stay unit (up to a 48 h stay), or admit to an in-patient ward [21, 22].

To assist with the implementation of this service reconfiguration, the National Acute Medicine Programme ‘categorised’ Irish hospitals into 4 generic hospital models, from the smaller Model 1 community/district hospitals to the largest Model 4 hospitals. The type of AMAU at each hospital was determined by the hospital’s model [see Additional file 1]. The Programme recognised that Units should be designed firstly around function, such as identifying and clarifying their role in the hospital’s acute services and specifying the patient groups to be assessed there, rather than form (e.g., physical layout and structure) and sites were given the flexibility to adapt the Units to suit local needs and resources [21]. This approach has been highlighted in Australia as being of significant importance in the performance of AMUs [23]. In addition to the establishment of these Units, the National Acute Medicine Programme identified four medical patient pathways - from ambulatory care through to care for complex patients requiring longer hospital stays - and recommended specific practice changes in each pathway [24, 25] [see Additional file 2].

While hospitals were not mandated to adopt this new framework for acute medical care, they were actively encouraged to do so. In 2010, when the NAMP model of care was published, there were eight acute public hospitals with an AMAU. Implementation started over the course of 2012 and 2013, with the last Unit opening in 2014. Currently there are 30 hospitals with an AMAU, representing over 88% of acute public hospitals. Seven out of nine model 4 hospitals, 16 of 17 model 3 hospitals and seven of eight model 2 hospitals have an operating AMAU [22].

### Understanding successful implementation of AMUs

#### Effectiveness of AMUs in improving patient outcomes

There is mixed evidence on the effectiveness of AMUs in improving patient care. Two recent reviews have expanded upon the initial systematic review conducted by Scott et al. in 2009 [26] and conclude that hospital length of stay, in-hospital mortality and 28-day readmission rates are reduced when AMUs are introduced into hospitals. However, the included studies were of moderate quality; the majority presented aggregate results (unadjusted for potential confounders), and relied on historical controls and ignored secular trends [17, 27]. A more recent systematic review by NICE assessed whether admission or assessment through an AMU (compared with direct admission to a general medical ward) increased hospital discharges, improved patient outcomes and hospital resource usage, and found that there is mixed evidence for the benefit of admission through an AMU [28]. With stricter inclusion and exclusion criteria, their review was limited to just three observational studies [29–31], which they classed as very low quality. Recognising the continuing growth in the area of Acute Medicine and the fact that over 90% of hospitals in the UK now have an AMU, the NICE committee felt that ongoing assessment of AMUs was crucial, especially in terms of adherence to standards and quality indicators, and called for higher quality research on the impact of AMUs, including measuring improvements in patient flow and reduced length of hospital stay [28].

#### Components of AMUs related to better outcomes

The heterogeneity of the AMU models studied in these effectiveness reviews, in terms of Unit organisation, consultant work patterns, ward round frequency, policies on length of stay, and admission criteria, and the fact that most studies have examined a single site, makes it hard to deduce *which* elements of the AMU are associated with better patient outcomes [17, 27]. The Society of Acute Medicine in the UK called for research to describe *what* features of an AMU contribute most to improved patients outcomes [20]. In response to this, Reid et al., conducted a second systematic review - this time to examine the evidence base on *how best* to deliver care in AMUs. They found limited evidence and a significant knowledge gap on the topic. The one component with consistent evidence of improved patient outcomes is the presence of a consultant for a sustained period [18]. This has been associated with a reduction of potentially avoidable admissions to hospital [8], reductions in mortality and 28-day readmission rates [32], and reduced length of hospital stay [14]. Hence, consultant presence is deemed a core component of AMUs worldwide [13, 15, 20, 33, 34] and the Royal College of Physicians in the UK have published recommendations on how to provide this consultant cover [35]. Vaughan et al., synthesised the literature on the benefits of a multidisciplinary team (MDT) in the acute medical setting on patient experience and clinical outcomes [36]. They found that there is a consistent, albeit methodologically flawed, body of evidence that supports MDT working in this setting. They highlight that the recent shift toward individualised care plans for patients, and the introduction of care bundles with specific interventions, necessitates a MDT approach to care. Whilst these care bundles and comprehensive care plans have not been extensively studied in the AMU setting, the literature supports the contentions that they are highly adaptable and promote MDT working, while certain components appear highly suitable for transfer into the AMU context [36].

#### Determinants of successful implementation of AMUs

There is a significant gap in the acute medicine literature concerning the factors influencing the implementation of these AMUs. To date, there are no published studies which have qualitatively examined the barriers and enablers to the establishment and embedding of these Units. The London Quality Standards programme which aimed to improve the quality of acute and emergency care, set out the minimum quality of care that patients with medical illnesses should expect when admitted to hospital. An evaluation of its implementation identified many barriers and enablers to adherence to standards in acute care [37], and it is likely that many of these will be of relevance to this study, given the similarity of programme objectives.

As other jurisdictions consider the expansion of AMUs [17], evaluating the recent, large scale, country-wide, implementation of Units into Irish hospitals provides an excellent opportunity to highlight the factors (contextual and others) which can facilitate or impede the implementation and impact of these Units on patient care.

### Approach to evaluation and conceptual frameworks

We will use a mixed methods approach (a multistage evaluation with an embedded explanatory sequential design) to examine whether the programme is achieving its desired outcomes, and how these outcomes are affected by the context within which the programme is operating [38].

The UK Medical Research Council (MRC) guidance on process evaluation of complex interventions will serve as the overarching framework [39, 40]. This framework recognizes that to inform policy and practice, we need to understand not only *whether* interventions work but *how* they were implemented, their causal mechanisms, and how effects differ from one context to another [39]. Programmes are frequently deemed to be ineffective, simply because they have not been implemented as planned [41, 42]. Therefore, evaluating *how well* a programme has been implemented is essential to understanding and interpreting an impact evaluation.

**Fig. 1:**
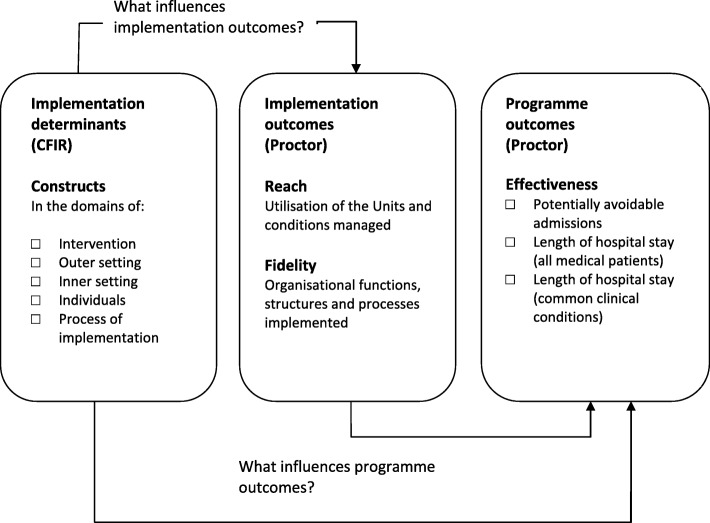
Conceptual approach to the evaluation of the National Acute Medicine Programme. Combining Damshroder’s ‘*Consolidated Framework for Implementation Research’* (CFIR) [43] and Proctor’s *‘Conceptual Model of Implementation research’* [44]

Damshroder’s *‘Consolidated Framework for Implementation Research’* (CFIR) [43] and Proctor’s ‘*Conceptual Model of Implementation research’* [44] will be used to understand the determinants of implementation and how they influence outcomes. The CFIR provides a comprehensive taxonomy of constructs that are likely to influence the implementation of complex programmes [43]. When using the CFIR in post-implementation evaluation studies, a focus on outcomes is essential and the meaningful use of the CFIR in this regard involves linking CFIR constructs (i.e., the determinants of implementation) to outcomes (both implementation & programme outcomes) [43, 45]. A recent systematic review by Kirk et al., categorising the empirical use of the framework, found a dearth of studies linking determinants of successful implementation to such outcomes [46]. Proctor et al., provide a model for distinguishing between implementation outcomes (e.g., adoption, reach and fidelity) and programme outcomes (e.g., service level outcomes - efficiency, effectiveness; patient level outcomes - satisfaction, quality-of-life) and highlights that a programme will not be effective if it is not implemented well [44]. We will use these frameworks to examine the hypothesized relationships depicted in Fig. 1.

## Methods

### Study aims

This study aims to evaluate the impact of Ireland’s National Acute Medicine Programme and identify the factors influencing its implementation and operation.

### Study objectives

1. Elicit the programme’s theories, and ‘mechanisms of change’ necessary to achieve the desired outcomes
2. Assess how the programme has been implemented across hospitals by measuring utilisation of the Units and documenting which organisational functions (i.e., structures, resources and processes) have been put in place to support the programme
3. Identify the factors (contextual and others) which have influenced the implementation of the programme and its outcomes
4. Determine whether the programme is achieving its outcomes and measure how well variation across sites can be ‘attributed’ to the level of implementation.

### Study design

**Fig. 2:**
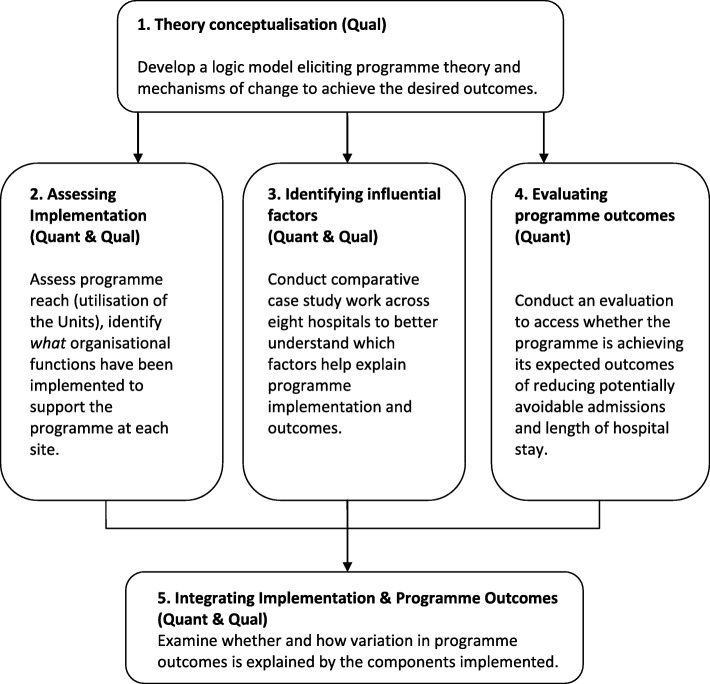
A multistage evaluation of the National Acute Medicine Programme

This multistage mixed methods study uses an explanatory sequential design whereby qualitative research will be undertaken to explain quantitative findings (see Table 1 and Fig. 2). In Stage 1, documentary analysis and data from expert interviews with the national programme team will be used to develop the programme’s logic model, specifying the underlying programme theory. In Stage 2, implementation effectiveness will be examined by using routine hospital administrative data to assess utilisation of the Units, and by conducting surveys to assess the organisational functions (i.e., structures, resources and processes) put in place at each site to support the Units. In Stage 3, comparative case study work will be conducted at eight sites to explore in detail the factors that have influenced the programme’s implementation and its ability to achieve desired outcomes. In Stage 4, routine administrative hospital data will be analysed again, this time to examine the impact of the programme in reducing length of stay of medical patients. In Stage 5, data from Stages1 to 4 will be integrated to examine how variation in programme outcomes across sites is explained by the level of implementation, and components implemented.

**Table 1:** Procedures and outputs for each stage of the evaluation

Stage	Procedures	Outputs
1. Theory Conceptualisation	1. Elicit the programme’s theory and depict it in a logic model using: a. Documentary review b. Key informant interviews c. Discussions with NAMP programme staff	1. Logic model depicting the programme’s mechanisms of change, desired outcomes, and programme components 2. Description of the organisational structures, processes and resources to be implemented
2. Assessing Implementation	1. Work with the NAMP team to design a survey for sites to assess ‘fidelity’ to the NAMP model of care based on the description of organisational structures, processes and resources be implemented from Stage 1 2. Measure programme reach - (Unit utilisation and conditions seen) using secondary administrative data	1. Quantification of what organisational functions have been implemented at each site 2. Measure of programme reach-utilisation of the Units and clinical conditions managed
3. Identifying factors influencing implementation and operation of the programme	1. Purposively sample eight sites based on their level of utilisation 2. Undertake semi-structured interviews with clinical staff at these sites 3. Import interview transcripts and documents into NVivo, code deductively using CFIR (and inductively) using Framework Analysis 4. Rate the CFIR constructs at each site to reflect their influence on implementation 5. Construct a matrix of cross case comparison; identify constructs that can possibly differentiate implementation	1. Descriptive memo of each case 2. Rating of the influence of each construct at each site 3. List of constructs that can successfully differentiate implementation
4. Evaluating impact	1. Clarify the expected programme outcomes and impact using the logic model 2. Create analytical datasets using secondary/administrative data 3. Estimate programme impact on reducing lengths of stay, and potentially avoidable admissions using interrupted time series	1. Estimation of programme effectiveness in reducing overnight emergency admissions for potentially avoidable medical conditions and reducing lengths of hospital stay and bed days used by medical patients
5. Integrating findings	1. Integrate qualitative findings from the each stage of the study	1. Comprehensive account of what has been implemented and what has been achieved and the factors that are influencing both of these

### Stage 1: Theory conceptualization

As suggested by the UK Medical Research Council guidance on process evaluation, unearthing the programme’s theory and depicting same in a logic model is a crucial first step in evaluating a complex intervention [40]. A logic model can be used to present both *process* and *impact theory* and is a replica of what the programme is intended to be which can then be analysed [40, 41]. It can be used for identifying the programme’s functions, activities and outputs to assess fidelity, and to understand how the programme interacts with the organisation’s structures and functions.

#### Data collection and analysis

**Fig. 3:**
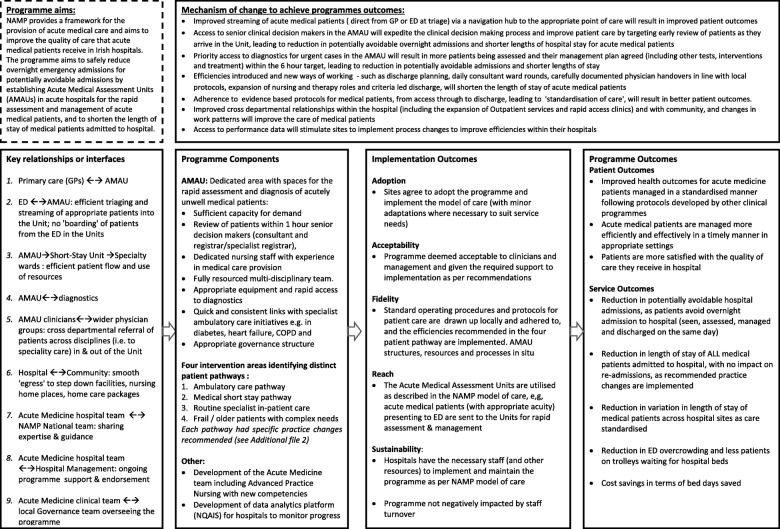
Logic model of the National Acute Medicine Programme

Following the guidance of Rossi et al. [41], a stepwise approach to eliciting the programme theory was taken, and this stage is completed. Rossi advises that to describe the theory embodied in an existing programme’s structure and operation, it is necessary that the evaluator work with stakeholders to draw out the theory that is represented in their actions and assumptions. Therefore, a logic model outlining the programme’s theory was developed by a combination of documentary review, key informant interviews, and in-person meetings with the NAMP team. Documents were reviewed to identify the underlying programme theory, the core components of the programme, the expected outcomes and the mechanisms as to how the programme expects to achieve these [47]. These included the national plan ‘Report of the National Acute Medicine Programme (2010), ‘standards’ and ‘guidance’ for AMUs in other jurisdictions [13, 15, 48] and published literature on their operation [18] and impact on patient care [17, 27, 28]. Key informant interviews were conducted with the NAMP team - physicians, nurses and allied health professionals with expertise in acute medicine, and programme managers - (past and present NAMP members, *n* = 6) and an initial group meeting (current members, n = 6) held to understand programme processes and how they could be influenced by the system into which they were introduced. A first draft of the model was developed in the format recommended by the Kellogg Foundation with emphasis not only on the programme’s outcomes and components but the mechanisms by which it expects to achieve these outcomes [49] and revised through face-to-face discussions with the national team [see Fig. 3].

### Stage 2. Assessing programme implementation

Studies in the UK and Australasia have shown considerable variation across hospitals in terms of compliance with recommendations on how care should be delivered in AMUs [50–52]. We are interested in examining whether the NAMP model of care has been implemented as designed. In our study, programme implementation will be assessed in terms of (i) ‘service utilisation’ (programme reach), defined by Rossi as *‘...the extent to which the intended targets actually receive programme services,* and (ii) the ‘organisational functions implemented’ again, defined by Rossi as *‘whether the programme’s actual activities and arrangements sufficiently approximate the intended ones*’ [41].

#### Data collection and analysis

Measuring the programme’s organisational functions focuses on *how well* the programme is organising its efforts and using its resources to accomplish the essential programme tasks [41]. The logic model will be used to develop a survey to collect data on the structure of the Unit (e,g, location, bed capacity, opening hours), resources (e.g., priority access to diagnostics, medical and nursing staff and workforce patterns), processes and procedures (e.g., mode of access to the Unit, referral pathway from ED/GP, patient profiles to be seen, escalation policy, return policy,) and changes in acute care throughout the hospital (e.g., improved patient streaming, integrated discharge planning, use of a common screening tool). Surveys will be completed at each site by the AMAU lead physician or Clinical Nurse Manager (CNM).

Service utilisation (programme reach) will be measured by examining the proportion of ‘emergency medical patients’ streamed through each Unit for 2017 and the case-mix and characteristics of these patients.

### Stage 3. Identifying factors that influence programme implementation and outcomes

To identify the factors which have influenced the implementation of the programme and its outcomes, we will conduct comparative case study work, using the approach by Yin which is suited to the complex nature of health services research, and allows for in-depth data gathering on organisational processes and programme impact [53, 54]. The purpose of this stage of the evaluation is to understand the factors that are influencing the level of utilisation of the Units in terms of the proportion of acute medical in-patients that are streamed through the AMAU, but also the factors that are influencing the programme’s ability to achieve its desired outcomes. Experience with the intervention, including participants’ perception of it and its compatibility with the hospital system will be explored in detail during this stage.

#### Selecting the sites for comparative case study research

Cases will be purposively sampled based on the level of utilisation identified during Stage 2, with four ‘high’ and four ‘low’ implementation sites selected. Sampling cases at either end of the implementation spectrum will allow us identify the factors that contribute to or hinder ‘successful’ implementation. This approach has been taken by Damshroder & Lowery in their study assessing implementation determinants for their propensity to distinguish between sites with high versus low implementation effectiveness [18].

#### Data collection at sites

We will conduct semi-structured interviews with health professionals (AMAU lead physician, clinical nurse manager for the AMAU, Assistant Director of Nursing for patient flow) to elicit information on the determinants of implementation [see Additional file 3 for list of CFIR constructs].

#### Analysing and interpreting the case studies

Data will be managed using NVivo 12. Qualitative data (interviews and documents) will be analysed using the Framework method which comprises five stages (familiarisation; identifying a thematic framework; indexing; charting; mapping and interpretation) [55, 56]. The CFIR framework will be applied as pre-defined deductive codes, however open coding will also be used to identify factors that do not fit within the definitions of CFIR constructs [57]. A case memo will be created for each of the eight sites, and constructs rated to reflect the magnitude of their influence on implementation, using the approach recommended by the authors of the CFIR framework [45]. A matrix will then be created listing the ratings for each construct for each site, and cross case comparison made between high and low implementation sites to identify patterns in ratings of the constructs that distinguish between high and low implementation effectiveness—i.e., constructs that were qualitatively correlated with implementation effectiveness [45].

### Stage 4. Evaluating programme impact

Outcomes were identified during the development of the logic model, which involved an examination of how AMUs are evaluated elsewhere. Programme effectiveness will be assessed by examining changes over time in (i) rates of potentially avoidable admissions (ii) lengths of hospital stay of medical patients (iii) lengths of hospital stay of potentially avoidable medical conditions.

#### Data collection and analysis

Programme outcome measures will be derived from Hospital In-Patient Enquiry (HIPE) which is an administrative database of all public hospital admissions in Ireland, including episodes of care in the AMAU. Programme impact will be estimated by comparing monthly data from 2009 to 2017, using interrupted time series regression (ITS) and ARIMA (autoregressive integrated moving average), accounting for secular and seasonal trends, and using the proportion of patients treated in the Units as a time varying covariate. Models will be run for individual AMAUs and for all hospitals combined. Several sensitivity analyses will be conducted including using length of stay truncated at 30 days, to account for the deficiencies in community services which can skew the average LOS.

### Stage 5. Integration of programme outcomes and programme implementation

To explain the variation in implementation and programme outcomes between sites, we will then construct a joint display table presenting the data for each site - the constructs that were identified as influential and the outcomes achieved - and examine patterns and inconsistencies across and between cases [58, 59] In this manner, the constructs which influenced implementation (Stage 3), the level of implementation (Stage 2) and the programme outcomes achieved (Stage 5), will be presented for each case in line with our conceptual framework from Fig. 1.

## Discussion

This protocol outlines a mixed methods study to evaluate whether the reconfiguration of acute medical care in hospitals, is effective in everyday practice. The study will examine the variation in implementation and effectiveness of Acute Medical Units from a national perspective, and be the first to comprehensively assess the factors that contribute to how well these Units are implemented and how well they perform. This work is timely as other jurisdictions consider the wide-scale introduction of Acute Medical Units [17]. It addresses the call for high quality research (including qualitative studies) to describe *which* features of Acute Medicine contribute most to its success [20]. The work of Reid et al., highlights the lack of research into the active ingredients of the AMU that contribute to its success and the clear gap in knowledge of how best to deliver care in the AMU [18, 27, 52, 60]. By examining variation in service and patient level outcomes in parallel to the organisational functions (e.g., structures, resources and processes of care), this study will assess the association between implementation of the AMU and outcomes achieved. The comparative case study will identify which components and processes contribute to improved outcomes and importantly, help decipher the factors that have influenced the successful establishment and operation of these Units. The results of this study will inform further refinement of the national programme and contribute to the design of more effective AMAUs.

Our study has some limitations. The fragmented health IT infrastructure in Irish hospitals, and the lack of a Unique Health Identifier, means we are unable to examine the trajectory of care received by patients streamed through the Units, and the impact on outcomes such as 30-day mortality, health services utilisation and quality of life. For this reason, we are examining the efficiency, effectiveness and timeliness of care [44] which are seen as ‘proximal’ outcomes upstream on the pathway to improved health outcomes [61]. We will examine changes in potentially avoidable admissions and lengths of hospital stay; the most common outcomes examined in previous studies evaluating the effectiveness of Acute Medical Units. We are also limited in our ability to examine indicators of performance. For example, we are unable to track - for all hospitals participating in the programme- the patient journey from ED through to the AMAU and the length of time spent on this pathway, which is an important indicator of the timeliness of patient care. Work is underway to address these IT shortcomings with the introduction of an Acute Floor Information System (AFIS), which will facilitate tracking of the patient journey and enhance the collection and reporting of these key performance indicators. A second limitation is the inherent risk of confounding that presents in observational studies of this nature. We have tried to minimise these risks by the use of robust statistical techniques such as interrupted time series analysis [62] at the individual hospital level, and the triangulation of various data sources to elicit a greater understanding of *how* the programme is resulting in improved outcomes.

This study has many strengths; most notably its explanatory sequential design which strengthens the validity of our findings on *what* influences implementation and *how* implementation leads to better outcomes. According to Creswell, combining statistical trends (quantitative date) with personal experiences (qualitative data), provides a better understanding of the research problem than either form of data alone [59]. Additionally, because complex interventions such as NAMP, tend to be highly context-specific in their effects, generalising the results of effect estimation for policy and practice requires more nuanced analyses of why these effects occur [63].

Second, programme and implementation theory will be used throughout; from the creation of the logic model which provides a blueprint of the programme to be analysed [40], to the use of the Consolidated Framework for Implementation Research [43] to guide data collection, measurement, coding, analysis and reporting of the findings of the comparative site work.

Third, we will use robust statistical methods to evaluate the performance and impact of these Units. Recent reviews of the literature on the effectiveness of these Units have highlighted the shortcomings in the research to date with many studies reporting effect estimates that have not taken into consideration potential biases (such as selection bias), confounding and underlying secular and seasonal trends [17, 27, 28]. We endeavour to minimise the influence of these on the effect estimates by using interrupted time series analysis and applying ARIMA modelling to estimate programme impact, adjusting for autocorrelation and seasonality [64–66].

Finally, the access to and collaboration with the national programme is a key strength of the study, as it facilities the co-development of the programme theory from the outset.

We expect the findings of this evaluation to be of interest to a wide audience given the growing need to demonstrate effectiveness of complex interventions. Findings on the mechanisms and contexts that optimise the implementation of this complex multi-faceted intervention will be useful to those developing and implementing other change programmes, especially given the growing realisation that failure to deliver effective services is largely attributable to the lack of knowledge on how best to implement and sustain these changes. This is a formative evaluation and the National team tasked with implementing and overseeing the programme are keenly interested in knowing what the determinants of a successful AMU are and how this information can be used to support sites struggling to reach full potential. Additionally, those in jurisdictions where the discipline of acute medicine is better developed and AMUs are well established, as well those in countries contemplating expansion of their AMUs, will be interested in the challenges that implementers face, and how context ‘interacts’ with and ‘shapes’ the programme being implemented.

## Supplementary information

**Additional file 1.** Description of AMAUs to be established by hospital model. Word document describing the Units.

**Additional file 2.** The four patient pathways specified by NAMP and the practice changes recommended. Word document (table format) describing the four patient pathways.

**Additional file 3.** Description of the five CFIR domains and constructs within each domain. Word document (table format) describing the CFIR constructs.

## Data Availability

The data that support the findings of this study are available from the corresponding author.

## Abbreviations

AM: Acute medicine
AMAU: Acute Medical Assessment Unit
AMU: Acute Medical Unit
CFIR: Consolidated Framework for Implementation Research
ED: Emergency Department
GP: General Practitioner
HSE: Health Service Executive
NAMP: National Acute Medicine Programmme
SSW: Short Stay Ward
