# Supplementary material for: A multistage mixed methods study protocol to evaluate the implementation and impact of a reconfiguration of acute medicine in Ireland’s hospitals
Source: BMC Health Serv Res. 2019 Oct 29;19:766. doi: 10.1186/s12913-019-4629-5 (PMC6819558; doi:10.1186/s12913-019-4629-5)
Supplement: Supplementary file 1 — Additional file 1. Description of AMAUs to be established by hospital model. Word document describing the Units. [file 12913_2019_4629_MOESM1_ESM.docx]

**Description of AMAUs to be established by hospital model**

The National Acute Medicine Programme ‘categorised’ Irish hospitals into 4 distinct models, providing a clear delineation of services provided within the constraints of available facilities, staff, resources and local factors. The level of service that can be safely provided at a hospital determines its hospital model, and therefore the assessment Unit ‘type’. Model 4 hospitals are larger teaching hospitals that accept undifferentiated acute medical patients and patients referred from other hospitals. Model 3 hospitals are larger hospitals that accept undifferentiated patients. Model 2 hospitals are smaller hospitals that accept differentiated patients; and Model 1 hospitals are community hospitals.

While guidance on the functioning, components and staffing of Unit by model type was provided, the National Acute Medicine Programme recognised that Units should be designed around function and not solely 'form' - an approach which has been highlighted in Australia as being of significant importance in the performance of AMUs– and therefore sites were given the flexibility to adapt the Units to suit local needs and resources. It was envisaged that Units in Model 4 hospitals would operate on a continuous 24/7 basis (contingent on appropriate staff and access to diagnostics), Model 3 Units would operate over a 12-24 hour period, 7 days a week depending on service need, and Model 2 Units would also be open 7 days a week but only from 8am to 8pm**. See over for further details.** Model 1 hospitals are community/district hospitals with sub-acute in-patient beds. These hospital do not have an ED,ICU, high dependency unit, coronary care unit or an AMU/AMAU/MAU.

Sites were asked to create and implement Standard Operating Procedures/Policies (SOPs) to standardise operations and processes within the Unit, including an escalation policy when there are no new assessment spaces in the Unit to assess new patients; clinical pathways for the management of medical conditions commonly seen in the AMAU in line with other National Clinical Programmes; criteria for the transfer of patients from the Emergency Department to the Units; and patient eligibility protocols including the specification of conditions NOT suitable for referral to the Unit.

Examples of AMU exclusion criteria from one Model 4 hospital include patients from the following categories: *requiring resuscitation or haemo-dynamically unstable; acute ST elevation MI; thrombolysis for stroke or myocardial infarction; surgical cases - abdominal pain, recent head injury (within 1 week), trauma; psychiatry cases, including alcohol detoxification; dermatology; oncology / haematology patients (undergoing / undergone active treatment in last 3 months); query DVT - proven DVTs are accepted- diagnostics performed in ED; back pain.*

**Function and operation of the Assessment Units by Hospital Model**

| **Hospital model**  **Description**  **Unit recommended** | **Brief programme guidance on function and operation of the Assessment Units** |
| --- | --- |
| **Model 4**  Larger, teaching hospitals that admit  undifferentiated acute medical patients and accept tertiary referrals  Acute Medical Unit  (AMAU and SSW) | Units should operate on a continuous 24/7 basis, dependent on the availability of medical, nursing and allied health care staff and services such as diagnostics available 24/7.  Ideally co-located with the ED in an acute floor model or at least in close proximity to the Emergency Department. Should be co-located with a Short Stay Ward/Unit, which is dedicated to the rapid turnaround of acute medical patients in whom the anticipated length of hospital stay is 72 hours or less. The SSW should function 24 hours a day, seven days a week and be co-located with the AAU to provide rapid disposition of patients from the AAU, and the potential or shared functionality with regards to medical, nursing and support staff as required. AMU physicians to have governance over the SSW beds.  Should have a designated lead consultant physician, clinical nurse manager and therapy lead. Hospitals should implement consultant rotas to suit resources including physician of the day/week model. Consultant time while in the AMU should be dedicated/protected. Ideally should be 6-8 physicians who work 80% in the Unit and SSW The AMU consultant physician is to be present on a continuous basis for a 12 hour period (8 am – 8 pm), weekdays and for 5 hours per day on weekends and public holidays. The consultant physician on-call for the hospital will manage the AMU out-of-hours.  Ideally patients will be referred directly from primary care but a proportion will be onward referrals from other sources including the ED, outpatients and other care settings, co-ordinated by a case manager. Patients presenting to hospital without GP referral will be seen in the first instance in the ED – not the AMU.  Upon arrival at the AMU, all patients should have nursing evaluation and Early Warning Score (EWS)/observations performed within 20 minutes. Initial medical assessment should be performed by a senior decision maker within 1 hour. Consultant physician review with a decision made to admit or discharge should be made within 6 hours.  The AMU will only admit patients for a short period for acute treatment and/or observation where the estimated length of stay is less than **48 hours**. Patients who require admission for longer than **48 hours** must move from this unit to an in-patient ward. |
| **Model 3**  Larger hospitals that accept undifferentiated patients  Acute Medical Assessment Unit | Operates as an AMU (as described above) with the following exceptions:   - Hours of operation may vary from 12 to 24 hours, 7 days a week, depending on service need - It does not have access to ring-fenced short stay medical beds   Units will have assessment beds in a defined area, ideally co-located with the ED.  Every AMAU should have a designated lead consultant physician, clinical nurse manager and therapy lead.  The Unit will see GP referred patients with the entire spectrum of acute medical conditions, some of whom may require urgent medical care. Patients presenting to hospital without GP referral will be seen in the first instance in ED – not the AMAU.  Upon arrival at the AMU, all patients should have nursing evaluation and Early Warning Score (EWS)/observations performed within 20 minutes. Initial medical assessment should be performed by a senior decision maker within 1 hour. Consultant physician review with a decision made to admit or discharge should be made within 6 hours. A decision regarding discharge/admission should be made within 6 hours and will be facilitated by dedicated radiology, laboratory and other services, including nursing, therapy professionals and medical social workers. In the event of discharge, the relevant GP will be informed (on the same day) of the decision together with all relevant clinical details and care plans.  Patients admitted to hospital from the AMAU will be to in-patient beds including specialist units (e.g. CCU, ICU, HDU, acute stroke unit). Patients who require category 3 or 3S ICU support will have guaranteed transfer to a Model 4 hospital. |
| **Model 2**  Smaller hospitals that accept differentiated patients  Medical Assessment Unit (MAU) | MAUs may be operational from 8am to 8pm, 7 days per week, depending on service need.  Units will have assessment beds in a defined area and serve a clinical support function.  Every MAU should have a designated lead consultant physician, who will be jointly appointed to the Model 2 and associated Model 3 or 4 hospital, a designated clinical nurse manager and assigned therapy resource.  The Unit will manage GP referred, differentiated medical patients who have a low risk of requiring full resuscitation. Only patients referred by a GP will be seen. GPs will refer low-risk medical patient (i.e. unlikely to require high intensity cardiopulmonary and/or neurological support) for assessment in the MAU during daytime hours. Patients with a significant risk of clinical deterioration should be referred to the associated Model 3 or 4 hospital.  A decision regarding discharge/admission should be made within 6 hours and will be facilitated by dedicated radiology, laboratory and other services, including nursing, therapy professionals and medical social workers.  Patients admitted to hospital from the MAU will be to in-patient beds in a Model 2 hospital. Patients who deteriorate unexpectedly will have guaranteed transfer to a Model 3/4 hospital. |
