# Supplementary material for: A multistage mixed methods study protocol to evaluate the implementation and impact of a reconfiguration of acute medicine in Ireland’s hospitals
Source: BMC Health Serv Res. 2019 Oct 29;19:766. doi: 10.1186/s12913-019-4629-5 (PMC6819558; doi:10.1186/s12913-019-4629-5)
Supplement: Supplementary file 2 — Additional file 2. The four patient pathways specified by NAMP and the practice changes recommended. Word document (table format) describing the four patient pathways. [file 12913_2019_4629_MOESM2_ESM.docx]

**NAMP: The four patient pathways and the changes to be implemented across each.**

**Pathway 1: Ambulatory care pathway**

**Assess and avoid admission in AMAU**

Patients receive safe and effective treatment in the Assessment Unit and are discharged on the same day.

- Establishment of adequate assessment area (AMAU) operating 12-24 hours, 7 days per week
- Development of clinical criteria for transfer between ED and AMAU
- Rapid access and diagnosis - access to senior decision maker within 1 hour
- Priority access to diagnostics and Health & Social Care Professionals (HSCP) assessment
- Quick and consistent links with specialist ambulatory care initiatives e.g. in diabetes, heart failure, acute coronary syndrome, asthma, COPD and epilepsy programmes.
- Rapid access to Outpatient clinics and scheduled follow up
- Liaison with discharge planner & relationship with community teams
- GP Liaison

**Pathway 2: Medical Short stay pathway**

**Short stay unit (1-2 nights)**

Developed for patients who require inpatient care but are not expected to stay longer than 1 or 2 nights.

**Pathway 3: Routine specialist inpatient care**

**Efficient** **processing of patients with length of stay between 3 - 14 days.**

These patients are admitted either directly to specialist medical wards from AMAU or via the Short-Stay Unit within 2 days of arrival.

**Pathway 4: Frailer and older patients with complex needs after discharge**

**Appropriate care and discharge of complex patients care pathway**

Frail older patients have complex care needs following discharge, and their discharge requirements must be identified early.

- Establishment of adequate short-stay unit for larger hospitals
- Access to *senior decision maker* within 12 hours of transfer from AMAU
- Continuous senior doctor supervisions (i.e. 7 days a week)
- Twice daily consultant ward rounds
- Access to prioritized diagnostics and HSCP assessment
- Carefully documented physician handovers in line with local protocols
- Documented management plan, inc target date of discharge, further diagnostics, interventions and necessary treatments
- Proactive case management, and integrated discharge planning
- Care formally handed over from the AMAU team to the Consultant Physician in line with local protocol
- Patients to be seen by their Consultant Physician with 12-16 hours of transfer to the ward
- Daily consultant ward rounds
- Daily review of medical patients on a board/ward round with senior doc
- Written medical and nursing care plans, inc. active discharge planning with planned dates of discharge
- Pro-active consideration for MDT assessment and intervention
- Weekend nurse/HSCP-facilitated discharges
- Development of clinical criteria to support flow to community hospitals within hospital groups
- Liaison with care givers and community
- Early assessment and identification of complex patients
- Proactive approach to identify the complex care needs of these patients when they are discharged
- Daily board rounds and consultant review
- Streaming to care of the elderly services where appropriate
- Proactive referral for MDT support
- Proactive MDT discharge planning and liaison with funding agencies for referral to community placements and supports
- Raise organisational awareness of:

the impact of extended lengths of stay on patient safety and,

the effectiveness of the hospital to manage capacity and demand

**Source:** Adapted from Royal College of Physicians of Ireland and Health Service Executive, *National Acute Medicine Programme. Key elements of the programme to deliver the best patient outcomes*. 2012. Available at: http://www.hse.ie/eng/services/publications/Clinical-Strategy-and-Programmes/National-Acute-Medicine-Programme.pdf

:
